# Address on Dental and Oral Surgery

**Published:** 1887-08

**Authors:** John S. Marshall

**Affiliations:** Chairman of the Section on Dental and Oral Surgery, Chicago, Ill.


### THE DENTAL REGISTER.

VOL. XLI.] AUGUST, 1887. [No. 8.

### COMMUNICATIONS.

### Address on Dental and Oral Surgery.

Delivered at the Thirty-eighth Annual Meeting of the American Medical Association, June, 1887.

BY JOHN S. MARSHALL, M.D.,

Chairman of the Section on Dental and Oral Surgery, Chicago, Ill.

The year which has just closed has been one of steady advancement in the department of dental and oral surgery, though I am unable to report any particularly new discoveries or brilliant achievements.

The investigations in the etiology of dental caries by Drs. Miller, of Berlin, Mills and Underwood, of London, and Black and others of our own country are still being pursued by these gentlemen, but no recent important announcements have been made. The opinion that the disease is caused by micro-organisms developed in the mouth is, however, rapidly gaining ground, for the conclusions reached by these investigators point very strongly in that direction.

A great deal has also been written during the year upon the treatment of pyorrhœa alveolaris, but very little has been added to the knowledge we already possessed of its etiology. The most notable improvement in methods of treatments is that introduced by Dr. Adair. This consists in applying to the roots of the teeth, after the concretions have been removed, a saturated solution of iodine in wood creasote, and then filling the pockets with tannic acid dissolved in glycerine, to form a protective dressing, and repeating this treatment once each day until the pockets are closed by new tissue. A method very similar to this was introduced by Dr. W. H. Atkinson some twenty years ago, and which at first met with very general adoption, but soon fell into disuse.

Since the introduction of Dr. Adair's remedies, many have called to mind those of Dr. Atkinson, which consisted of four formulas, viz.:

1. Chloride of zinc and re-sublimed iodine, equal parts rubbed together.

2. Saturated solution of iodine in alcohol.

3. Saturated solution of iodine in wood creasote.

4. Pure wood creasote. Later he substituted caustic potash and crystalized carbolic acid, rubbed to a paste in a warm mortar, and which are now being used with much better success than formerly, doubtless owing to the fact that the disease itself is better understood, and consequently the remedies needed for its successful treatment.

New instruments for various purposes and of unique forms have been invented almost without number, consequently we can not take space to enumerate them. Some are real improvements over the forms already in general use, but the greater number are of no real particular value, except to furnish employment for the instrument makers.

The subject which has seemed to occupy the minds of the dental practitioners more perhaps than any other, has been that of *pulpless teeth* and their treatment. Not that there has been any change in the mode of treatment, for this seems to be as nearly perfect as it is possible with our present knowledge to make it; but rather because they have been called upon, by one of our leading medical journals, to defend their practice. The journal in question condemned the practice of treating and retaining in the jaws teeth which were diseased or dead, upon evidence, which to the dental specialist at least, seemed very meagre indeed, and then drew the conclusion, that as a result of such practice, "nervous diseases about the head were becoming alarmingly common."[1] While the editor in the same number of the

1 Medical Record, Vol. 26, No. 14, page 374.

journal calls attention to the article from which the above is quoted, and says it "furnishes additional evidence of the perils of tooth saving."[2] These articles immediately brought out several vigorous protests from the dentists, but the discussion of the subject was cut short by the editor, as it doubtless seemed likely to become too voluminous for the pages of his journal. But the vital question as to whether *dead teeth* could be retained in the jaws, be made useful and at the same time inoffensive to the general health of the individual, did not receive the attention that its importance seemed to deserve. The term *dead teeth* is usually applied to teeth which from disease or traumatism have lost the vitality of their pulps, and not in its broader sense, for that would imply also a loss of vitality in the cementum and the pericementum. Teeth in this condition would give rise to irritation and acute inflammatory processes of the surrounding tissues, and like all irritating foreign substances be soon expelled from the economy.

The term *pulpless teeth*—one in common use among dentists—better describes the condition of the teeth under discussion, and hereafter when referring to this condition I shall confine myself to the use of this term. The teeth, as is usually understood, receive their nutrition and sensation from two distinct sources, viz.: through the pulp which fills the central canal, and the pericementum which covers the root. The pulp is largely composed of blood vessels and nerve fibres, which are continuations of the maxillary arteries and nerves, while the blood vessels and nerve fibres of the pericementum, with the exception of a few small twigs from the above mentioned sources, are derived entirely from the gingival arteries and nerves.

The pulp gives vitality to the dentine and the enamel, which make up about four-fifths of the bulk of the tooth, but which contains only from 13 to 18 per cent of organic matter when taken together. The pericementum gives vitality to the remaining portion, viz.: the cementum, which is analagous to bone and contains from 30 to 40 per cent of organic material, and it is through the pericementum that the teeth have their attachment to the alveolus.

2 Idem 379.

The death of the pulp *per se* has no effect upon the functions or vitality of the cementum or the pericementum, for there is no direct communication between them, and experience teaches that these tissues may perform their office in a normal manner for an indefinite period after the destruction of the pulp, provided the dentine has been treated antiseptically, and the central canal hermetically closed at the apex of the root.

In advanced life there is a tendency to obliteration of the pulp by peripheral calcification, a process similar to, if not identical with, the formation of the dentine. Many times the pulp will be found reduced to a mere thread, and in others the bulbous portion is entirely calcified. The functions of the pulp in these cases are more or less impaired or destroyed, but no discernible effect is produced either upon the functions of the cementum or pericementum.

There is, however, a form of calcification of the pulp known as interstitial, in which nodules of secondary dentine or osteo-dentine are found in the body of the organ, which frequently gives rise to severe neuralgia of the face and head, and reflex irritations resulting in diseases of the eyes and ears.

In the articles referred to, the cases cited to substantiate the conclusions at which the authors arrived, presented no satisfactory evidence that would indicate the teeth as pulpless, or that they were the seat of alveolar abscess or pericementitis; in fact, the evidence was strongly in favor of the supposition that the reflex irritation mentioned was caused by an irritated and inflamed condition of the pulps, the result of an advanced stage of caries. The question which very naturally arose in the minds of all intelligent dental practitioners who had carefully read the articles, was whether the gentlemen who criticised the methods of treatment pursued by dentists in the treatment of pulpless or diseased teeth, and which have been the means of saving thousands and tens of thousands of teeth in a useful condition, some for ten, twenty, thirty, and even forty years, without detriment to the general health, were competent to criticise; and whether after all they really knew the difference between a simple case of pulpitis and one of pericementitis, or any thing whatever of their symtomalogy.

There is no doubt that teeth which are diseased from alveolar abscess (the result of the death of the pulp), accumulations of salivary calculus about the necks and roots; pericementitis or irritation and inflammation of the pulp from caries, etc., occasionally gives rise to acute neuralgias of the head and face, and reflex nervous irritation, resulting in functional derangement and organic diseases of the eyes and ears, but that this is by any means common I do not believe, nor is the radical treatment insisted on by these writers, viz., the extraction of all suspicious teeth, the only or the best treatment to be recommended, for the greater proportion of such cases are amenable to scientific treatment. The dentist who would practice such wholesale and ruthless destruction of the teeth would not be worthy the name of dentist, for it would be contrary to the natural impulses of humanity and against all sound surgical principles. The educated dentist has been taught that it is his mission to *save* and not to *destroy* the teeth; and all his energies are brought to bear in this direction, while extraction is to be practiced as a *dernier resort.*

Pulpless teeth which have been treated by removing the devitalized or putrescing pulp, the central canal and the canaliculi of the dentine rendered antiseptic, the apical foramen hermetically plugged with an indestructible and non-irritating material, and the canal and cavity of decay properly filled, will, in nine cases out of ten, give rise to no untoward symptoms, and remain in the jaws indefinitely in a serviceable condition; the truth of which no doubt can be vouched for from personal experience by many gentlemen of the Association. It occasionally happens, however, that a pulpless tooth, from the small size of its central canal or a sharp curvature of its root, can not be successfully treated in the manner described; these will in many instances, sooner or later, give trouble from alveolar abscess; but because a few unfavorable cases can not be treated with success is not a good reason for discarding the treatment.

Inflammatory conditions of the pulp, especially the subacute and chronic forms, very much oftener result in facial neuralgia and reflex troubles of the eyes and ears, than do pulpless teeth which have been treated by the method just described, "although no appreciable irritation may be experienced by the patient in the affected tooth."[3] Such cases can be cured just as readily and permanently by the devitalization of the pulp, followed by the usual treatment for pulpless teeth, as by the heroic measure of extraction, and be infinitely better for the patient. A sufficient number of cases might be presented, gathered from the published records, to satisfy any fair minded member of the association of the truth of these statements; but the time at my disposal will not permit me to do so.

One of the authors of the articles referred to "regards the treatment of pulpless teeth by the practice in vogue, as the reverse of procedures founded on well established surgical principles, since the stopping of the natural outlet for the escape of putrescing products from portions of the pulp left remaining in the canal, and dental canaliculi through the exterior part of the tooth itself make their passage into the tissues underneath unavoidable. The diversion of the drainage must be of questionable propriety in many instances, since the tissues about the roots of the dead teeth are liable to become infiltrated with the products of decomposition, the absorption of which, when slowly formed, is much more liable to contaminate the system than the discharge of pus into the mouth from an alveolar abscess."[4] The argument looks plausible, and if the statements upon which it is founded were correct, it would be unanswerable, but unfortunately for the writer and his theory they are not. He speaks of closing "the natural outlet for the escape of putrescent products," etc., from the tooth. Which is "the natural outlet," the apical foramen or the cavity of decay? I answer, that depends upon circumstances. When there is no communication between the central canal and the cavity of decay the apical foramen is the *only* outlet; when the cavity of decay communicates with the central canal then the cavity of decay becomes the *natural* outlet. Other things being equal an abscess left to itself always chooses the shortest and quickest route—the one offering the least resistance—to the point of outlet. But it is the duty of the surgeon

3 Medical Record, Vol. xxvi, page 525.
4 Medical Record, Vol. xxvi, page 525.

to cut short the process by giving exit to such accumulations of pus or putrescent material, wherever located, at the earliest possible moment, and thus shorten the period of suffering and remove the danger from septic poisoning. The same obligation rests upon the dentist.

When there is no communication between the cavity of decay and the central canal through which discharge can take place, gases are formed by the processes of decomposition, and septic material is forced through the only outlet, the apical foramen.

The root of the tooth being imbedded in a bony alveolus and surrounded by the tissues of the face and mouth, septic products discharged at this point not only immediately set up inflammatory action, resulting in acute alveolar abscess, but from its location and the character of the tissues to be penetrated in seeking an outlet, several days are required to accomplish it, and this, unless operative measures are employed for its cure, soon becomes chronic and more or less constantly discharges as the case may be either into the mouth, the antrum of Highmore, the nasal fossa, or through the external tissues of the face, and which not infrequently, if left to run its course, terminates in other and more serious complications. Such cases, however, can in a majority of instances be *arrested* in their course, and in still a greater number *prevented* if taken in time, by giving exit to the contents of the pulp canal by simply drilling an opening into the canal through the cavity of decay, or other convenient location, and removing the septic material. This is the treatment pursued by reputable dentists. Is it a proceeding contrary to sound surgical principles?

When the apical foramen has been properly closed there is usually no more trouble with alveolar abscesses, for septic materials are prevented from coming in contact with the surrounding tissues. Is such practice "the reverse of well established surgical principles?" I think not. It is nature's own method of trying to prevent a recurrence of the disease, for it not infrequently happens that the apical foramen in pulpless teeth after a time becomes completely closed by a deposition of secondary cementum, doubtless the result of this previous irritation of the pericemental membrane. Can we go far astray when we follow such teaching? Are we not constantly trying to understaud the processes by which nature restores a diseased organ to health, and the laws which govern them, that we may profit by the knowledge, and so shape our treatment as not to antagonize them, but rather prove helpful? This is what the dentist is trying to do by the method pursued in treating pulpless teeth. This is not empiricism, but enlightened, rational practice.

Furthermore, when the central canal has been thoroughly cleansed by removing the remains of the devitalized pulp, the apical foramen sealed, the cavity properly dried, the dentinal canaliculi rendered antiseptic by appropriate treatment, the central canal and cavity of decay so plugged as to exclude air and moisture, it is hardly possible for "products of decomposition to be formed in the canal or canaliculi, pass into the tissues beyond and be absorbed."

The antiseptics generally used are wood creasote, carbolic acid, and bichloride of mercury, either of which will prevent decomposition; but when as in this method of treatment, the effect of excluding the air and moisture are added to the power of the antiseptic remedy, it seems almost impossible for decomposition to take place. It is admitted that in those cases which have not received appropriate treatment such conditions do exist; but even in these extraction is not called for, as it is yet possible to place a good proportion of these in a healthy condition.

In behalf of our specialty, I, therefore, enter a most earnest protest against the principles set forth in these articles, in so far as they attempt to prejudice the profession against a line of treatment that has stood the test of time and proved so signally successful in the hands of dental practitioners, and hope the members of the Association will not be inclined to accept such conclusions as a final settlement of the question, but carefully investigate every case of disease of the eyes and the ears, and neuralgias of the head and face, in relation to the influeuce of pulpless and diseased teeth in producing such conditions, and instead of advising the immediate extraction of the suspected teeth, the patient be placed in the hands of a skillful dentist for appropriate treatment.

If such practices should become general we should not be confronted with the statement, that as a result of the practice of dentists in treating pulpless teeth, "nervous diseases about the head were becoming alarmingly common." If the principles which underlie the practice of dentistry could be better understood by the general practitioner, there would be fewer people in the world with endentulous jaws, and consequently more people with robust digestions; for it is a lamentable fact that with many the value of the teeth in promoting the general health is generally under-estimated, and patients are advised to submit to the extraction of teeth that could be successfully cured of disease and made useful for years. The only way in which this end can be accomplished is through the medical colleges. This Association a few years ago recognized scientific dentistry as a department or specialy of medicine by the organization of a separate section. This has done much already to stimulate the young men entering the ranks of dentistry to take a full medical education, and is resulting in a demand for broader and more comprehensive teaching in the dental schools of the sciences upon which rational medicine and surgery are based. What is most needed, however, at the present time, in view of the subject presented in this address, is that the medical colleges of our country should each institute a professorship or lectureship on dental surgery, through which all future graduates shall receive at least a theoretical knowledge of dental and oral diseases and their treatment. The possession of such knowledge would place the general practitioners in position to correctly advise their patients with regard to the care and treatment of their teeth, be the means of preventing a great amount of suffering to their patients and annoyance to themselves from inability to correctly diagnose such diseases, as well as to check the wholesale destruction of organs so necessary to the preservation of the functions of digestion and a robust condition of health.

---

Another occupation is to be improved for men. Bellevue Hospital, New York, is promised a training school for male nurses—a rich Californian having given $80,000 towards putting it on a solid footing.

